# Ecotoxicology of Polymetallic Nodule Seabed Mining: The Effects of Cobalt and Nickel on Phytoplankton Growth and Pigment Concentration

**DOI:** 10.3390/toxics11121005

**Published:** 2023-12-08

**Authors:** Rimei Ou, Hao Huang, Xuebao He, Shuangshuang Lin, Danyun Ou, Weiwen Li, Jinli Qiu, Lei Wang

**Affiliations:** 1 Key Laboratory of Marine Ecological Conservation and Restoration, Third Institute of Oceanography, Ministry of Natural Resources, Xiamen 361005, China; m200200605@st.shou.edu.cn (R.O.); huanghao@tio.org.cn (H.H.); lin941200@163.com (S.L.); oudanyun@tio.org.cn (D.O.); liweiwen@tio.org.cn (W.L.); qiujinli@tio.org.cn (J.Q.); 2 Laboratory of Marine Biodiversity, Third Institute of Oceanography, Ministry of Natural Resources, Xiamen 361005, China; hexuebao@tio.org.cn; 3 College of Chemistry, Chemical Engineering & Environmental Science, Minnan Normal University, Zhangzhou 363000, China

**Keywords:** *Skeletonema costatum*, *Prorocentrum donghaiense*, metal toxicity, physiological response, environmental impact

## Abstract

In order to improve the understanding of the environmental impacts of polymetallic nodule mining, ecotoxicological studies were conducted on the growth of model phytoplankton species *Skeletonema costatum* and *Prorocentrum donghaiense* using cobalt and nickel. This study evaluated various physiological and ecological indicators, such as cell proliferation, chlorophyll *a*, pigments, total protein, and antioxidant enzyme markers. The results show that the introduction of low amounts of cobalt or nickel increased the growth rate of phytoplankton. The phytoplankton benefited from low concentrations of cobalt and nickel stress. The increased protein levels and decreased activity of antioxidant enzymes considerably impacted physiological responses during the promotion of cell abundance. High concentrations of cobalt or nickel resulted in decreased light-absorbing pigments, increased photoprotective pigments, an inactive chlorophyll content, decreased total proteins, and maximal antioxidant enzyme activity in phytoplankton. Throughout the experiment, both the phytoplankton protein and enzyme activity declined with prolonged stress, and the cells underwent age-induced damage. Thus, seabed mining’s repercussions on phytoplankton could result in both short-term growth promotion and long-term damage. These consequences depend on the impurity concentrations infiltrating the water, their duration, and the organism’s physiological responses.

## 1. Introduction

The depletion of terrestrial minerals has led to an escalating interest in seabed mineral deposits driven by the increasing demand for minerals and metals. Polymetallic nodules (PMNs), which are a source of strategic mineral reserves with the potential for commercial mining, comprise various critical elements such as nickel (Ni), copper (Cu), manganese (Mn), cobalt (Co), molybdenum (Mo), titanium (Ti) and lithium (Li). Co and Ni are the most economically valuable, particularly for the electric vehicles sector [1,2]. Despite their economic significance, PMN seabed mining results in inevitable environmental impacts. Previous and current research has primarily focused on the seabed ecosystem, including consequences such as habitat fragmentation, a loss of biodiversity, plume dispersion, and particle deposition [3,4,5].

Research into the environmental impacts of the pelagic layer, particularly the euphotic layer, is limited [6,7]. However, seabed mining poses a significant risk of diffusion and the contamination of surface-level metals [8]. In 2022, The Metals Company (TMC) conducted the inaugural trial of a deep-sea mining system in the Clarion Clipperton Zone (CCZ) situated in the eastern Pacific Ocean. During the process of separation on the ship, sediment and nodule fragments are discharged from the cyclone separator and subsequently spilled onto the surface of the ocean from the deck “https://metals.co/cyclone-separator-overflow/” (20 May 2023). Sediment and nodule fragments contain metallic elements and nutrients that can have a bearing on phytoplankton in the euphotic zone, either promoting or hindering their growth. This is a highly convoluted matter, and the specific impact depends on the concentration and endurance of the contaminants [9].

Metals can have harmful impacts on ecosystems due to their persistent, toxic, and bio-accumulative traits [10]. The general order of trace elements in phytoplankton, considering their overall relative cellular abundance, is Fe ≈ Zn > Mn ≈ Ni ≈ Cu > Co ≈ Cd [11,12,13]. In general, metals are toxic to phytoplankton at higher concentrations [14,15]. Metals can promote or restrain phytoplankton growth by affecting physiological factors such as proteins, enzyme activities, fatty acids, glycerol, and pigments [16,17]. Co and Ni play vital roles in the functioning of organisms but are recognized as emerging contaminants not only in coastal areas but also in open ocean settings [18,19,20,21]. Cobalt toxicity arises from haem oxidation, the blocking of inorganic calcium channels, cytotoxicity, and genotoxicity [22]. Studies on these metals have recently focused on environmental risk assessments [21], bio-accumulation, and toxic responses in phytoplankton [23]. Phytoplankton assimilate metals such as Co and Ni into their cells as micronutrients, which are essential for enzyme functions [11,12,24,25]. Information regarding the harmful impacts of Co and Ni on phytoplankton and their potential consequences on phytoplankton production is a matter of environmental importance. It is crucial to evaluate the toxicity of Co and Ni by employing bioassays on vulnerable marine organisms. The present investigation employs the model species *Skeletonema costatum* and *Prorocentrum donghaiense* to conduct an experimental ecological analysis. The aim of this study was to investigate the environmental impacts of deep-sea mining via the physiological and ecological effects of the diffusion of cobalt, nickel, and other metals from polymetallic nodules in seabed mining on phytoplankton growth and photosynthetic pigment composition. This study could provide a reference for the environmental impact assessment of polymetallic nodule seabed mining.

## 2. Materials and Methods

### 2.1. Strains and Design of Experiments

#### 2.1.1. Strains

*S. costatum* and *P. donghaiense* were from the State Key Laboratory of Marine Environmental Science, Xiamen University. The experiments were carried out after expanded culture with an f/2 culture medium (Leading tec for Ocean Science Co., Ltd., Shanghai, China) [26]. Seawater was first filtered using a 0.22 μm Pall^®^ AcroPak capsule and then sterilized in an autoclave at 121 °C for 15 min. After cooling down to an ambient temperature, the f/2 culture medium was added to the water in aseptic conditions. Following this, phytoplankton were inoculated into 2.4 L PETG media bottles with a square shape manufactured by Nalgene™. Under optimum conditions, the bottles were then placed in a 20 °C incubator, which was maintained at the same temperature with a light intensity of 7000 lux, a light/dark cycle ratio of 12 h:12 h, and shaking once a day.

The culture was centrifuged at 3500 rpm for 10 min, and the resulting algal cells were cultivated until they reached a density that was suitable for formal experimentation. As the f/2 medium formula lacked nickel, a phytoplankton expansion was conducted directly in the nickel-only experiment. In the cobalt-only experiment, all the phytoplankton were inoculated into a sterilized f/2 medium without cobalt for domestication until they reached a suitable density to conduct formal experiments.

#### 2.1.2. Design of Experiments

In this study, the treatment group without added cobalt and nickel was used as the control group. Three parallel incubation units were set up for each concentration gradient in the cobalt and nickel treatment groups. Pre-experiments revealed that *S. costatum* was less tolerant to cobalt and nickel compared to *P. donghaiense*, and the concentration gradients for the addition of cobalt and nickel were determined. In addition, taking the concentrations of these two elements leached from polymetallic nodule debris into account, the concentration gradients for the cobalt and nickel treatment groups were set as follows: for *S. costatum*, the cobalt treatment groups were set at 5, 50, and 500 μg/L, and the nickel treatment groups were set at 0.5, 2, and 5 μg/L. For *P. donghaiense*, the cobalt treatment groups were set at 30, 300, and 3000 μg/L, and the nickel treatment groups were set at 3, 30, and 300 μg/L. The cobalt and nickel solutions were in a NiSO_4_ and CoCl_2_ medium, respectively (Leading tec for Ocean Science Co., Ltd., Shanghai, China). Each experimental unit used a Nalgene™ 2.4 L PETG bottle.

### 2.2. Parameters

#### 2.2.1. Cell Abundance

The water in the incubation was adequately shaken, and then an appropriate amount was taken to the centrifuge tube and fixed with 10% of Lugol’s Solution (Beijing Solarbio Science & Technology Co., Ltd., Beijing, China). The abundance of phytoplankton cells was observed daily using a microscope. The growth curves of the cells were determined at different concentrations and compared with the control group to observe these differences.

#### 2.2.2. Chlorophyll *a*

The incubation water was filtered through Advantec^TM^ 25 mm diameter GF-75 (pore-size is 0.3 µm) glass fiber filters using Pall^®^ MicroFunnel filter funnels with a vacuum pressure of no more than 0.02 MPa. After filtration, the filter was stored at −80 °C until analyzed in the laboratory.

Chlorophyll *a* was determined using fluorescence analysis [27]. After the filter was extracted in 90% acetone solution in the dark and at −20 °C for 24 h, it was determined using a Turner Designs Trilogy fluorometer (Turner Designs, Sunnyvale, CA, USA). The measurement of chlorophyll *a* concentration was performed on a spectrofluorometer with the excitation and emission wavelengths set at 430 and 670 nm, respectively.

#### 2.2.3. Pigments

Seawater samples were filtered through Advantec^TM^ 25 mm diameter GF-75 (pore-size is 0.3 µm) glass fiber filters. These filters were then immediately frozen by storage at −80 °C until analyses using high-performance liquid chromatography (HPLC). In the laboratory, the frozen filters were extracted in 3 mL 90% HPLC grade acetone and placed in a refrigerator at 4 °C for 1.5 h. We used the reverse-phase HPLC method as described by [28]. Pigment separations were achieved using a 3.5 μm Eclipse XDB C_8_ column (100 × 4.6 mm; Agilent Technologies) connected to a Shimadzu LC-20 AT/Prominence HPLC system. Qualitative and quantitative analyses were confirmed using the standards manufactured by the Danish Hydraulic Institute Water and Environment, Hørsholm, Denmark. Chlorophyll *a*, fucoxanthin, chlorophyll *c*_2_, diadinoxanthin, alloxanthin, and pheophorbide were the main pigments of *S. costatum*. For *P. donghaiense*, the pigments included consisted of chlorophyll *a*, chlorophyll *c*_2_, peridinin, and antheraxanthin.

#### 2.2.4. Protein and Antioxidant Enzyme Markers

An appropriate amount of the culture sample was collected in a centrifuge tube and centrifuged at 4 °C and 4500 rpm for 10 min; the supernatant was discarded, 3 mL of phosphate-buffered solution (PBS) was added and transferred to a 5 mL centrifuge tube; this was centrifuged at 4 °C and 4500 rpm for 10 min before discarding the supernatant, adding 2 mL of PBS solution, and then the cells were disrupted by an ultrasonic crusher for 6 min. Finally, the disrupted sample was centrifuged at 4 °C and 4500 rpm for 10 min, and the supernatant was collected. Total protein (TP), superoxide dismutase (SOD), catalase (CAT), peroxidase (POD), trace-reduced glutathione peroxidase (GSH), and malondialdehyde (MDA) were determined using assay kits purchased from Nanjing Jiancheng Bioengineering Institute. The enzyme activities were determined according to the instructions, including the biuret method for the TP, WST-1 method for the SOD, the visible light method for the CAT, a colorimetric method for POD and GSH, and the TBA method for MDA.

#### 2.2.5. Metal Concentration

Water samples were filtered through a 0.22 μm syringe filter and transferred into 2 mL centrifuge tubes from each culture bottle. The tubes were subsequently stored in a 4 °C freezer. For water sample pretreatment, 1 mL of thawed water samples containing heavy metal ions for testing were added to a 15 mL centrifuge tube with ultrapure water. We measured the exact concentrations of cobalt (Co), nickel (Ni), iron (Fe), manganese (Mn), and copper (Cu) according to the method combining the isotope dilution with standard additions [29] and using the Agilent inductively coupled plasma mass spectrometer (ICP-MS7700x). The Quality Assurance/Quality Control (QA/QC) procedures followed PARCC principles, including precision, accuracy, representativeness, completeness, and comparability [30].

### 2.3. Data Analysis

The lack of an observed effect concentration (NOEC) and the lowest observed effect concentration (LOEC) were calculated according to the OECD Guidelines for the Testing of Chemicals [31] using the one-tail Dunnett’s procedure [32]. The NOEC and LOEC for the cell abundance, HPLC chlorophyll *a*, and the total protein were calculated as cell growth, photosynthetic pigments, and enzyme physiology, respectively.

The impact of various additives on phytoplankton growth was assessed utilizing one-way ANOVA, followed by the independent t-test. Statistically significant results were recognized at *p* < 0.05. All statistical analyses were conducted using IBM SPSS Statistics 26.0, whereas Origin 2022 (OriginLab Corporation, Northampton, MA, USA) was used for the column chart statistical analysis. All statistical analyses were conducted using IBM SPSS Statistics 26.0, whereas Origin 2022 (OriginLab Corporation, Northampton, MA, USA) was used for the column chart statistical analysis.

## 3. Results

### 3.1. NOEC and LOEC

The NOEC and LOEC after 120 h for cell abundance, chlorophyll *a*, and total protein of *S. costatum* and *P. donghaiense* were highly variable (Table 1). In both *S. costatum* and *P. donghaiense*, the NOEC and LOEC of each parameter were significantly higher under cobalt stress than under nickel stress. For example, the cell abundance inhibition of *S. costatum* under cobalt stress was 14.140 μg/L in NOEC and 26.077 μg/L in LOEC, which were higher than that of the values under nickel stress at 3.771 μg/L in NOEC and 5.505 μg/L in LOEC. *S. costatum* had a significantly lower NOEC and LOEC for all parameters compared to *P. donghaiense*, except for the two species under cobalt stress, for which the total protein NOEC values under cobalt stress were 45.374 for *S. costatum* and 42.854 for *P. donghaiense*. For NOEC and LOEC compared to each other, the difference between the two in cell abundance was slightly greater than chlorophyll *a*, while the difference between NOEC and LOEC for the total protein was the closest. This indicated that metal toxicity stimulates enzyme activity and protein synthesis, which, in turn, affects pigment synthesis and cell growth.

### 3.2. Cobalt Toxic Effects

#### 3.2.1. Cell Abundance and Chlorophyll *a* Concentration

**Figure 1:**
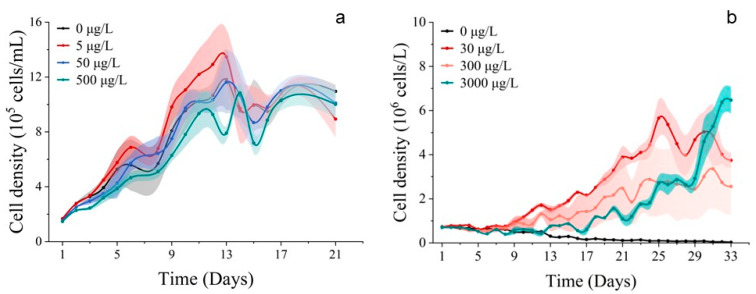
Population growth curves of phytoplankton. (**a**) Exposed for 21 d to different concentrations of Co^2+^ on *S. costatum*. (**b**) Exposed for 33 d to different concentrations of Co^2+^ on *P. donghaiense*. Mean value standard error bars (*n* = 3). The shadow area denoted the standard deviation.

**Figure 2:**
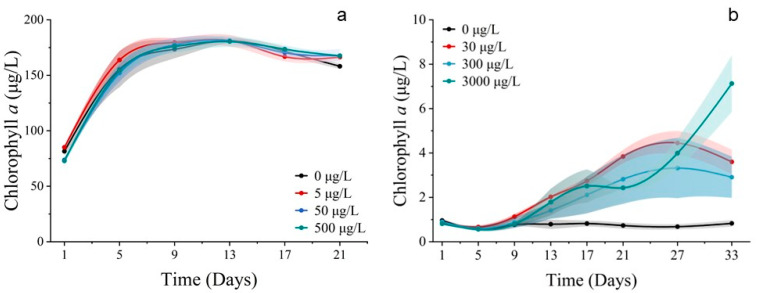
Concentrations of chlorophyll *a* of phytoplankton. (**a**) Exposed for 21 d to different concentrations of Co^2+^ on *S. costatum*. (**b**) Exposed for 33 d to different concentrations of Co^2+^ on *P. donghaiense*. Mean value standard error bars (*n* = 3). The shadow area denoted the standard deviation.

Population growth trends were observed for *S. costatum* and *P. donghaiense* when exposed to cobalt. In the 500 μg/L group, the cell abundance of *S. costatum* was inhibited, promoted in the 5 μg/L group, and unaffected in the 50 μg/L group (Figure 1a). The cell abundance of *P. donghaiense* was promoted by all Co^2+^ treatments, but the cell abundance of the 30 μg/L group was higher than that of the 300 μg/L treatment group, 3000 μg/L treatment group, and control group (0 μg/L). It is noteworthy that the cell abundance of the 3000 μg/L group consistently exceeded that of the 300 μg/L and 30 μg/L treatment groups at 25 d and 31 d (Figure 1b). The chlorophyll *a* concentration of *S. costatum* and *P. donghaiense* exposed to Co^2+^ closely resembled their respective population growth curves (Figure 2).

#### 3.2.2. Pigments

- Pigments of *S. costatum*

**Figure 3:**
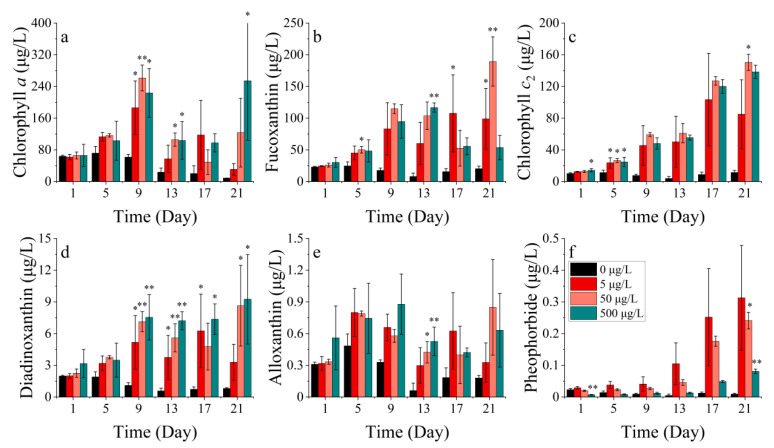
Pigment concentrations of (**a**) chlorophyll *a*, (**b**) fucoxanthin, (**c**) chlorophyll *c*_2_, (**d**) diadinoxanthin, (**e**) alloxanthin and (**f**) pheophorbide at *S. costatum* for different concentrations of cobalt. * indicates a significant difference between the experimental addition group and control group on the same day (*p* < 0.05); ** indicates a highly significant difference between the experimental additional group and the control group on the same day (*p* < 0.01).

The *S. costatum* comprises six photosynthetic pigments, including chlorophyll *a*, fucoxanthin, chlorophyll *c*_2_, diadinoxanthin, alloxanthin, and pheophorbide. The levels of chlorophyll *a*, fucoxanthin, chlorophyll *c*_2_, diadinoxanthin, alloxanthin, and pheophorbide differed significantly between the treatments (*p* < 0.05). At cobalt concentrations of 5, 50, and 500 μg/L, all pigment concentrations were significantly elevated compared to the control group (Figure 3). Chlorophyll *a* was significantly lower when exposed to all Co^2+^ concentrations compared to the control group after 9 days and according to LSD’s test. The concentrations in the four treatments were 61.710, 186.311, 261.289, and 223.718 μg/L, respectively. On the 13th day of the experiment, chlorophyll *a* concentration decreased in all groups. However, the concentrations of chlorophyll *a* in the 50 and 500 μg/L cobalt concentration treatments (105.836 and 103.676 μg/L, respectively) were still significantly higher than that of the control group (23.763 μg/L) (*p* < 0.05). On day 21, the concentration of cobalt increased to its peak value of 254.209 μg/L after reaching a trough and was significantly higher than the control group’s concentration of 8.784 μg/L (*p* < 0.05) (Figure 3a).

Fucoxanthin exhibited different performances over time under varying cobalt concentrations (Figure 3b). The concentration of fucoxanthin in four treatments (0, 5, 50, 500 μg/L) reached peak values on days 5, 17, 21, and 13 of the experiment, respectively, with the highest concentrations of 24.696, 107.627, 189.353, and 116.666 μg/L. The concentrations were significantly higher in three cobalt treatments than in the control group (*p* < 0.05). When the concentration of cobalt was at 0, 5, and 50 μg/L, the concentration of fucoxanthin initially increased, then decreased, and later increased again after reaching a minimum point. However, when the cobalt concentration was at 500 μg/L, the fucoxanthin concentration gradually declined after reaching the peak on the 13th day.

Significant differences (*p* < 0.05) were observed between treatments for chlorophyll *c*_2_ (Figure 3c). The chlorophyll *c*_2_ concentration showed an increasing trend when cobalt concentrations of 5, 50, and 500 μg/L were used. Specifically, on day 5, the concentration of chlorophyll *c*_2_ in the treatment groups with cobalt concentrations of 5, 50, and 500 μg/L (23.679, 26.572, and 24.793 μg/L) was significantly higher than in the control group (11.296 μg/L), with increases of 109.623%, 135.233%, and 119.478%, respectively. By day 21, the concentration of chlorophyll *c*_2_ for the four treatments were 11.443, 85.030, 150.397, and 138.360 μg/L, with the concentration increasing by 643.054%, 1214.279%, and 1109.094% in the three cobalt treatments when compared to the control group. Additionally, the treatment group with a 50 μg/L cobalt concentration had the highest concentration of chlorophyll *c*_2_. The results indicated that cobalt stress had a significant impact on the synthesis of chlorophyll *c*_2_ with the concentration range of 5–500 μg/L promoting its synthesis. However, the highest synthesis rate was observed at a concentration of 50 μg/L cobalt. The diadinoxanthin concentration peaked at day 1, 17, 21, and 21 in the four treatments (0, 5, 50, 500 μg/L), respectively, with maximum concentrations of 1.987, 6.257, 8.645, and 9.257 μg/L, respectively (Figure 3d). The three cobalt addition groups exceeded the control group significantly (*p* < 0.05). Thus, when the cobalt concentration was added at 5 μg/L, the diadinoxanthin concentration gradually increased and later decreased. When the cobalt addition concentration was 50 and 500 μg/L, the diadinoxanthin concentration trend initially increased and then decreased, reaching a minimum before increasing again. Throughout most of the experiment, the concentrations of diadinoxanthin in these two treatments were higher than those in treatments with a low cobalt concentration addition.

Overall, the variation in the alloxanthin concentration in each treatment group started increasing before decreasing and then increasing again (Figure 3e). On the 9th day of the experiment, the concentration of alloxanthin in each treatment group (5, 50, 500 μg/L) was higher than that in the control group with an increase of 65.12%, 63.06%, and 53.00%, respectively. On the 21st day of the experiment, the concentrations of alloxanthin in the higher cobalt groups (50, 500 μg/L) were higher than the other two groups. The variation in the alloxanthin concentration, as a protective pigment, indicated that the addition of cobalt may require the synthesis of higher levels of alloxanthin by *S. costatum* to protect against peroxidation, and the higher cobalt concentration resulted in a higher and longer-lasting peroxidation. For pheophorbide, there were significant differences (*p* < 0.05) between the groups, and the variation in pheophorbide concentration showed a consistent increase in the cobalt treatment groups (Figure 3f). In the initial phase, although the pheophorbide concentration in the 500 μg/L cobalt treatment group was significantly lower than that of the control group, which accounted only 27.78% compared to that of the control group, on the 21st day of the experiment, the pheophorbide concentration was ranked as 5 μg/L > 50 μg/L > 500 μg/L > control, and the contents were 0.313, 0.241, 0.081 and 0.009 μg/L, respectively. This indicated that the synthesis of pheophorbide was significantly affected by cobalt stress.

- Pigments of P. donghaiense

**Figure 4:**
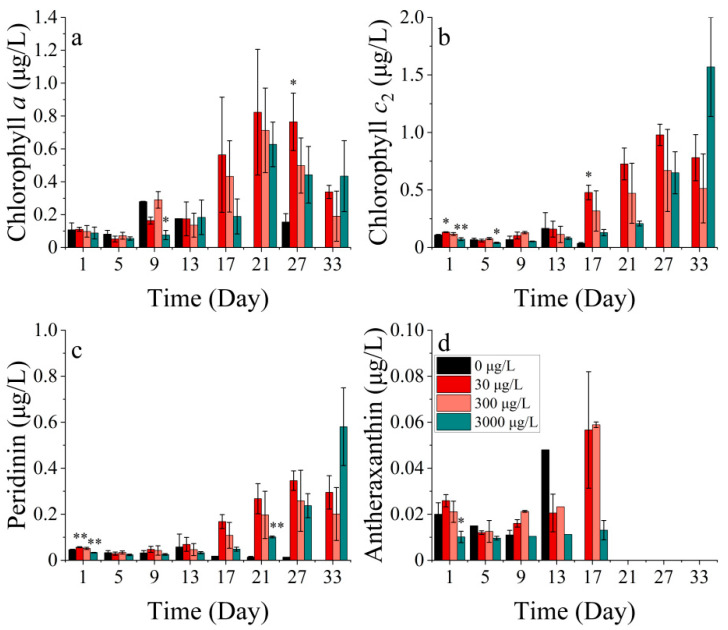
Pigment concentrations of (**a**) chlorophyll *a*, (**b**) chlorophyll *c*_2_, (**c**) peridinin, and (**d**) antheraxanthin on *P. donghaiense* in different concentrations of cobalt. * indicates a significant difference between the experimental addition group and the control group on the same day (*p* < 0.05); ** indicates a highly significant difference between the experimental addition group and the control group on the same day (*p* < 0.01).

The *P. donghaiense* contains four main photosynthetic pigments, including chlorophyll *a*, chlorophyll *c*_2_, peridinin, and antheraxanthin, where peridinin is the characteristic pigment of diatoms (Figure 4).

The concentrations of chlorophyll *a*, chlorophyll *c*_2_, peridinin, and antheraxanthin showed significant differences among the treatments (*p* < 0.05). Chlorophyll *a* concentration was not significantly different between the groups during the first 13 days of the experiment (Figure 4a). By contrast, the chlorophyll *c*_2_ concentration was significantly lower in the 30 μg/L cobalt-treated group on the first and fifth days of the experiment (*p* < 0.05) (Figure 4b). The concentrations of chlorophyll *a* and chlorophyll *c*_2_ in the 30 μg/L cobalt-treated group were significantly higher than those in the other groups on days 27 and 17 (*p* < 0.05), whereas the differences between the other groups for these two pigments were not significant after 13 days. Peridinin is a light-absorbing pigment, while antheraxanthin functions as a photoprotective pigment. In all cobalt concentration treatments, the synthesis of photoprotective pigments was enhanced. The highest synthesis efficiency was observed in the 30 μg/L treatment, followed by the 300 μg/L treatment. Over the 27-day experiment, the synthesis efficiency increased gradually from the lowest to the highest in the 3000 μg/L treatment (Figure 4c). The antheraxanthin levels were not detectable in any of the groups after 21 days. Prior to the 21st day period, the pigment concentrations in the treatments of 30 and 300 μg/L were noted to be higher than the other two treatments (Figure 4d). The concentration of antheraxanthin, as a light-harvesting carotenoid, followed an opposite trend to that of chlorophyll *a*. This pattern was also seen in the present study when the concentration of antheraxanthin declined rapidly after peaking, while chlorophyll *a* showed a rapid increase.

#### 3.2.3. Protein and Antioxidant Enzyme Markers

**Figure 5:**
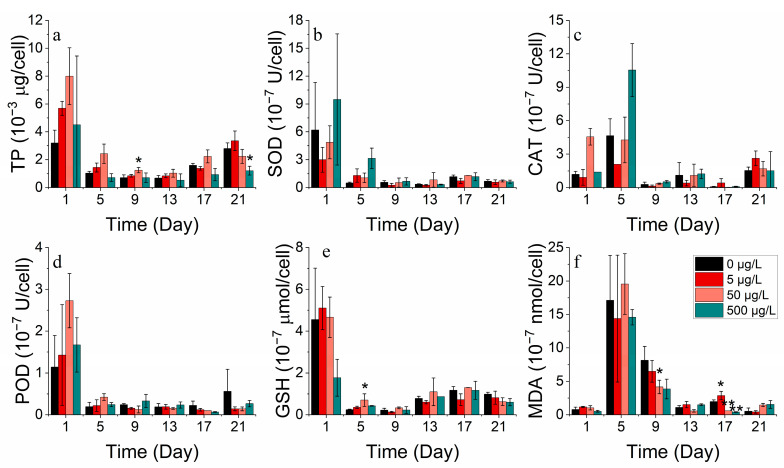
Enzyme concentrations of (**a**) TP, (**b**) SOD, (**c**) CAT, (**d**) POD, (**e**) GSH and (**f**) MDA on *S. costatum* in different concentrations of cobalt. * and ** indicate significant and highly significant differences between the experimental addition group and the control group on the same day, respectively (*p* < 0.05, *p* < 0.01).

SOD, CAT, POD, and GSH are significant indicators of phytoplankton’s response to environmental stress. At optimal concentrations, cobalt enhances the total protein production. However, excessive cobalt concentrations lead to a damaged protein structure or restricted protein activity. The addition of cobalt at lower concentrations (0–50 μg/L) was found to be advantageous for producing total protein in *S. costatum* in contrast to higher concentrations (500 μg/L) (Figure 5a). They were also found to increase at higher concentrations (500 μg/L) while the MDA concentration decreased. The results suggest that, at higher concentrations of cobalt, phytoplankton produces more antioxidant enzymes, protecting the cell membrane from peroxidative damage [33]. Antioxidant enzyme markers decreased after reaching their peak during the lag phase, implying cellular deactivation as stress continued to increase (Figure 5b–f).

**Figure 6:**
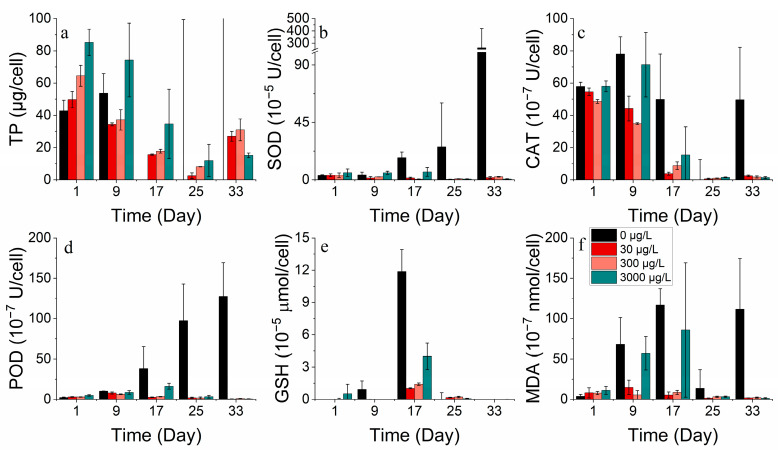
Enzyme concentrations of (**a**) TP, (**b**) SOD, (**c**) CAT, (**d**) POD, (**e**) GSH and (**f**) MDA on *P. donghaiense* in different concentrations of cobalt.

Cobalt enhanced the overall protein production of *P. donghaiense*. However, protein activity decreased with an increase in the incubation duration. The values of the enzymes SOD, CAT, POD, and GSH exhibited higher values in the groups with no addition and the highest concentration addition (3000 μg/L) compared to the medium addition concentration. Meanwhile, the enzyme activity levels were greater in the 0 μg/L group than in the 3000 μg/L group. The mortalities of *P. donghaiense* could not be avoided, possibly due to its high reliance on cobalt. This cobalt deprivation drove *P. donghaiense* to produce more antioxidant enzymes, defending cell membranes from peroxidative damage. However, adding cobalt concentrations ranging from 30 to 3000 μg/L stimulated the growth of *P. donghaiense* (Figure 6).

#### 3.2.4. Metal Absorption

Cobalt affects various physiological and ecological indicators in phytoplankton, including cell abundance, biomass, pigments, and enzyme activity. However, it cannot be solely attributed to the impact of cobalt, as phytoplankton growth is governed by a combination of metal elements. The order of dissolved metals in the water was identified as Fe > Mn > Cu > Ni > Co.

**Figure 7:**
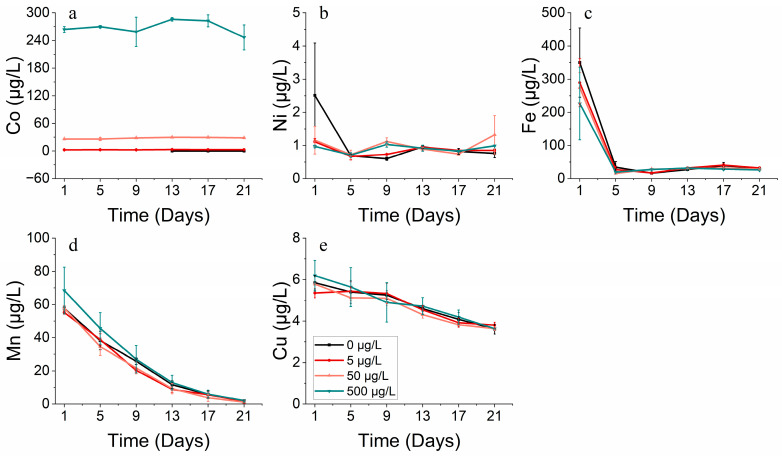
Metal concentrations of (**a**) cobalt, (**b**) nickel, (**c**) iron, (**d**) manganese and (**e**) copper on *S. costatum* in different concentrations of cobalt.

**Figure 8:**
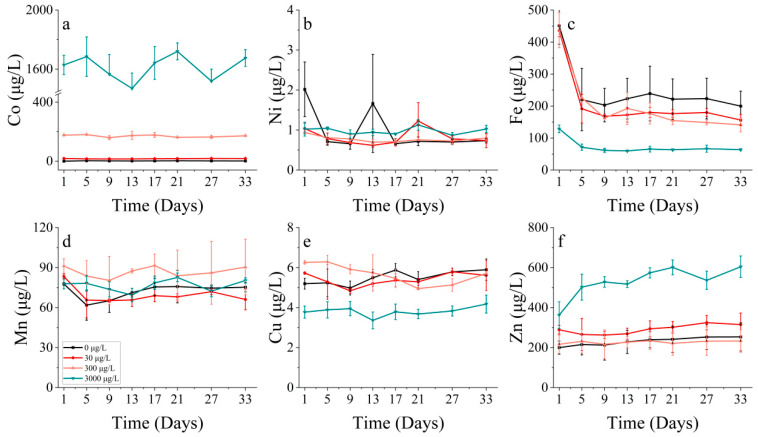
Metal concentrations of (a) cobalt, (b) nickel, (c) iron, (d) manganese, (e) copper and (f) zinc on *P. donghaiense* in different concentrations of cobalt.

The growth of *S. costatum* was hindered under high levels of cobalt stress, perhaps due to its co-interaction with other metals like Fe, Mn, and Cu. Different from the condition that produced a continuous reduction in the Mn and Cu concentration, the Fe concentration was at a relatively stable level and even increased slightly from the 9th day. As both carbon reduction and nitrogen reduction, the two primary systems that provide energy for photosynthesis, rely on iron-containing substances, it is evident that the concentration of iron (Fe) significantly impacts the growth of phytoplankton (Figure 7c and Figure 8c). For *S. costatum*, it seemed that Fe only had an effect during the lag phase (within the initial 5 days) of the experiment, while Mn and Cu produced an effect throughout the experiment. This permitted the ongoing intake of nutrients while Fe was absorbed at low levels (Figure 7c–e). Nonetheless, it appears that the phytoplankton maintained adequate levels of growth in the latter half of the experiment, primarily due to the influence of Mn (Figure 7d).

Concerning *P. donghaiense*, it appeared that Fe only played a major role during the initial lag phase of the experiment (within the first five days). Furthermore, the concentration of Fe also decreased until the 9th day, although there was no significant change in the Fe concentration after the 9th day (Figure 8c). At this juncture, cell abundance still increased in all the treatments where cobalt was added, apart from the control group. There seemed to be no distinct trend in the Mn uptake by *P. donghaiense* in different treatments in contrast to *S. costatum* (Figure 8d).

### 3.3. Nickel Toxic Effects

#### 3.3.1. Cell Abundance and Chlorophyll *a* Concentration

**Figure 9:**
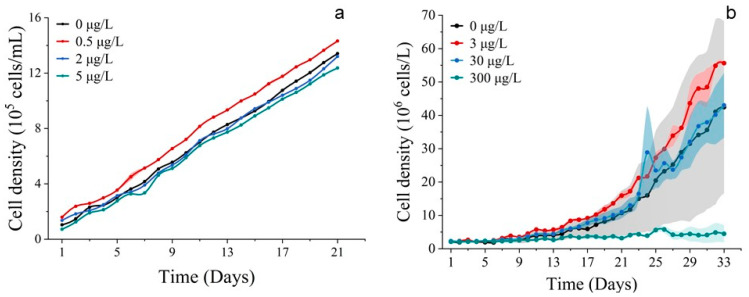
Population growth curves of phytoplankton. (**a**) Exposed for 21 d to different concentrations of nickel on *S. costatum*. (**b**) Exposed for 33 d to different concentrations of Ni ^2+^ on *P. donghaiense*. Mean values standard error bars (*n* = 3). The shadow area denoted the standard deviation.

**Figure 10:**
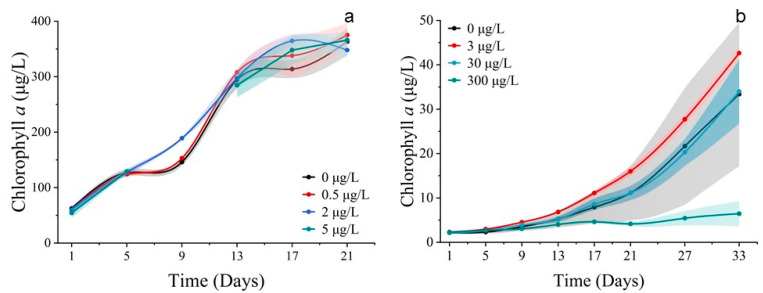
Concentrations of chlorophyll *a* of phytoplankton. (**a**) Exposed for 21 d to different concentrations of Ni ^2+^ on *S. costatum*. (**b**) Exposed for 33 d to different concentrations of nickel on *P. donghaiense*. Mean value standard error bars (*n* = 3). The shadow area denoted the standard deviation.

Lower concentrations of nickel (0.5 μg/L) increased the cell abundance of *S. costatum*, while higher concentrations (2 μg/L, 5 μg/L) reduced *S. costatum* cell abundance, with the inhibitory effect appearing greater in the 5 μg/L group compared to the 2 μg/L group (Figure 9a). The cell abundance of *P. donghaiense* was reduced in the 300 μg/L group, increased in the 3 μg/L group, and remained unaffected in the 30 μg/L group (Figure 9a). The chlorophyll *a* concentration in *S. costatum* and *P. donghaiense*, exposed to nickel, exhibited a similarity with the population growth curve (Figure 10).

#### 3.3.2. Pigments

**Figure 11:**
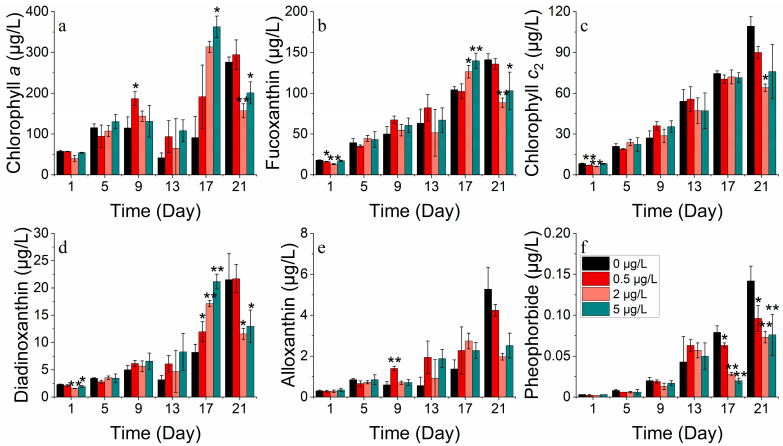
Pigment concentrations of (**a**) chlorophyll *a*, (**b**) fucoxanthin, (**c**) chlorophyll *c*_2_, (**d**) diadinoxanthin, (**e**) alloxanthin and (**f**) pheophorbide on *S. costatum* in different concentrations of nickel. * and ** indicates significant and highly significant differences between the experimental addition group and the control group on the same day, respectively (*p* < 0.05, *p* < 0.01).

Alterations in fucoxanthin concentration under various nickel stresses indicated that lower nickel concentrations (0, 0.5 μg/L) were more conducive to light absorption than higher concentrations (2, 5 μg/L). The long-term alterations in the diadinoxanthin and alloxanthin concentrations indicated that the concentrations of the three nickel treatments were remarkably lower than those of the control group. The addition of nickel at lower concentrations (0 and 0.5 μg/L) resulted in higher levels of photoprotective pigment synthesis on *S. costatum* compared to higher concentrations (2 and 5 μg/L). This has given rise to the suggestion that a certain concentration of nickel addition can diminish the risk of peroxidation reactions (Figure 11).

**Figure 12:**
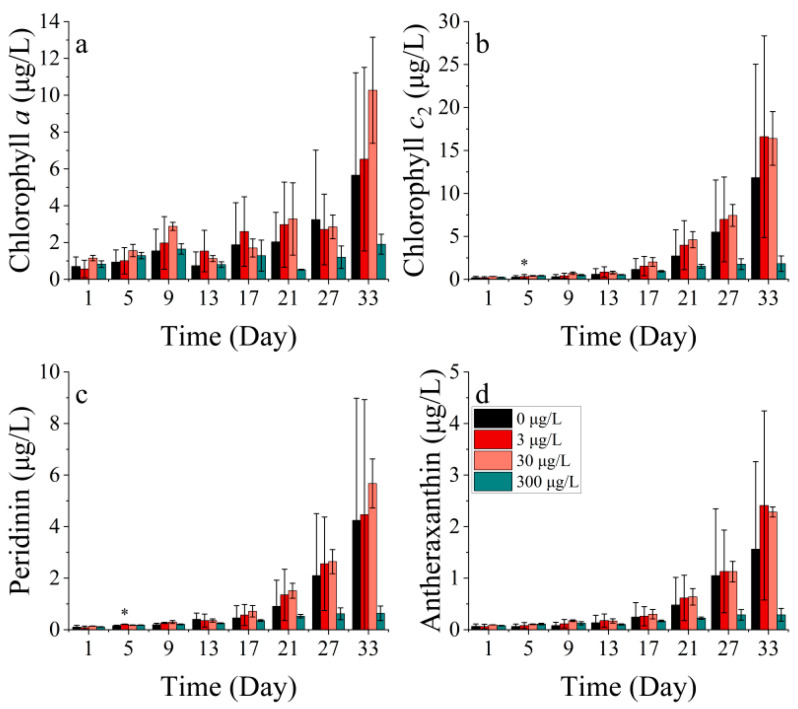
Pigment concentrations of (**a**) chlorophyll *a*, (**b**) chlorophyll *c_2_*, (**c**) peridinin, and (**d**) antheraxanthin at *P. donghaiense* in different concentrations of nickel. * indicates a significant difference between the experimental addition group and the control group on the same day (*p* < 0.05).

The levels of chlorophyll *a*, chlorophyll *c*_2_, peridinin, and antheraxanthin in *P. donghaiense* differed significantly across the treatments (*p* < 0.05). Chlorophyll *a* exhibited a bimodal trend over 33 days, with the peaks occurring at 9 and 33 days, respectively. There was no significant difference in the chlorophyll *a* concentration between the treatment groups compared to the control. (Figure 12a). Chlorophyll *c*_2_, peridinin, and antheraxanthin in *P. donghaiense* exhibited resemblances and showed an overall upward trend. Pigment concentrations followed the sequence 30 μg/L > 3 μg/L > 0 μg/L > 300 μg/L in all four treatments. Chlorophyll *c*_2_ and peridinin exhibited significant differences in the 3 μg/L cobalt addition treatment compared to the control group after 5 days, with lower and higher levels respective to each pigment (*p* < 0.05). The inhibition of all four pigments was observed at the highest nickel concentration of 300 μg/L (Figure 12b–d).

#### 3.3.3. Protein and Antioxidant Enzyme Markers

**Figure 13:**
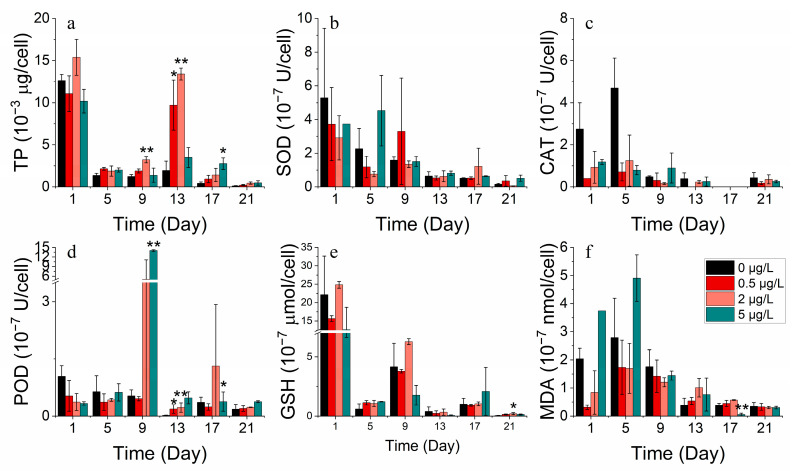
Enzyme concentrations of (**a**) TP, (**b**) SOD, (**c**) CAT, (**d**) POD, (**e**) GSH and (**f**) MDA on *S. costatum* in different concentrations of nickel. * and ** indicate significant and highly significant differences between the experimental addition group and the control group on the same day, respectively (*p* < 0.05, *p* < 0.01).

For *S. costatum*, total protein concentrations decreased, then increased, and subsequently decreased in all the nickel concentration treatment groups (Figure 13a). Almost all nickel groups showed higher total protein concentrations compared to the control group, indicating that nickel addition facilitated protein production. For a period, the 0.5 μg/L and 2 μg/L nickel treatment groups promoted total protein synthesis on the 9th and 13th day and also stimulated POD activity compared to the control. Cell membrane damage was also less severe at lower concentrations. However, the addition of higher nickel concentrations resulted in a higher increase in antioxidant enzyme activity to counteract the peroxidative effects of heavy metals (Figure 13b–f).

**Figure 14:**
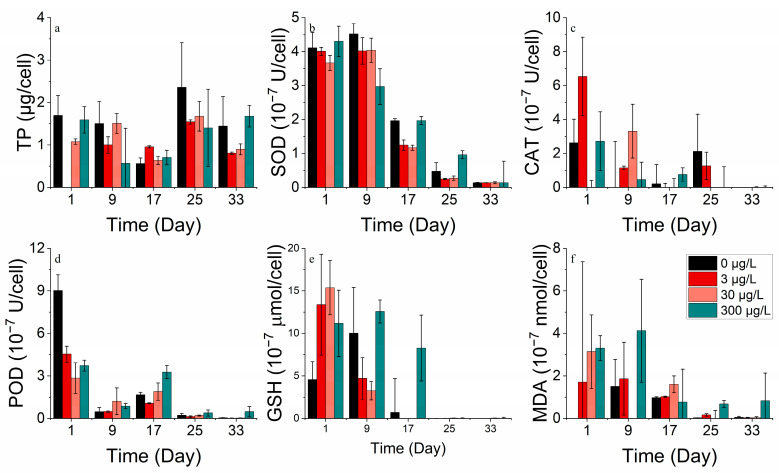
Enzyme concentrations of (**a**) TP, (**b**) SOD, (**c**) CAT, (**d**) POD, (**e**) GSH and (**f**) MDA on *P. donghaiense* in different concentrations of nickel.

The total protein concentration in all nickel addition treatment groups of *P. donghaiense* exhibited an initial decline, followed by an increase, and then another decrease (Figure 14a). There was no significant difference in the total protein between the treatment groups compared to the control. Moreover, the enzyme activities of SOD, CAT, POD, GSH, and MDA decreased progressively during the experiment. After 17 days, the enzyme activities of the group with the highest nickel addition (300 μg/L) surpassed those of the other groups (0, 3, 30 μg/L). It is worth noting that as time passed, the phytoplankton cells became more damaged and stressed, leading to a decrease in both protein and enzyme activity (Figure 13 and Figure 14).

#### 3.3.4. Metal Absorption

**Figure 15:**
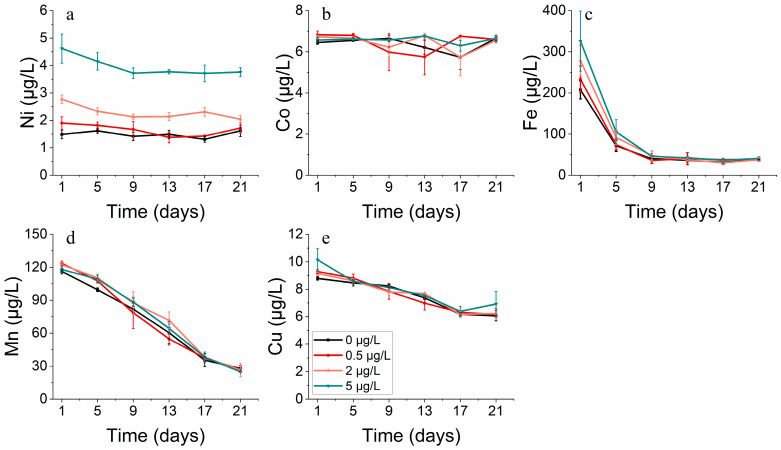
Metal concentrations of (**a**) nickel, (**b**) cobalt, (**c**) iron, (**d**) manganese and (**e**) copper on *S. costatum* in different concentrations of nickel.

There is a correlation among elements like Ni, Fe, and Mn. An increased Ni concentration hinders Fe uptake, and the addition of Ni affects Mn absorption. While phytoplankton absorbed Ni to some extent, *S. costatum* only absorbed Ni during the first half of the experiment for the highest Ni addition treatment. During the second half, the growth of *S. costatum* seemed to be due to the effects of Mn (Figure 15).

**Figure 16:**
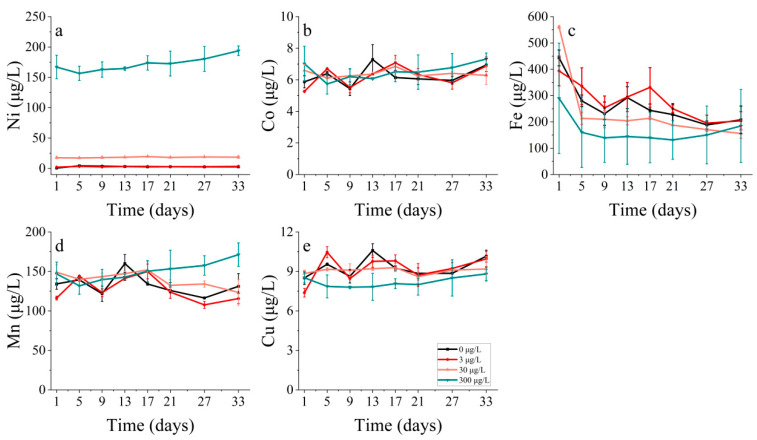
Metal concentrations of (**a**) nickel, (**b**) cobalt, (**c**) iron, (**d**) manganese and (**e**) copper on *P. donghaiense* in different concentrations of nickel.

The Fe concentrations in *S. costatum* displayed a positive correlation with increasing nickel concentrations. By contrast, the relationship between the Ni concentration and Fe concentration in *P. donghaiense* was more intricate. Specifically, the lowest Fe concentration was observed with the highest Ni concentration (300 μg/L) addition treatment, and the remaining three treatments showed a gradual decrease in Fe concentration over 5 days, with the highest Fe concentration observed for the 30 μg/L group (Figure 16c). There seemed to be no discernible trend in the uptake of Mn and Cu by *P. donghaiense* under the different treatments (Figure 16d,e).

## 4. Discussion

### 4.1. Potential Role for Co/Ni in Coregulating Phytoplankton Growth

The NOEC and LOEC for phytoplankton growth stress using cobalt and nickel are relatively rarely reported in previous studies. For the diatoms *Odontella mobiliensis* and *Coscinodiscus centralis* under cobalt stress, the 96 h NOEC was 170 μg/L and 370 μg/L, and the LOEC were 290 μg/L and 640 μg/L, respectively. By contrast, the 96 h NOEC and LOEC of *S. costatum* under selenium stress was 540 μg/L and 940 μg/L, respectively [34]. Clearly, the order of magnitude was higher compared to the 120 h NOEC and LOEC of the present results. For the green algae, *Chlorella vulgaris*, and *Pseudokirchneriella subcapitata*, the NOEC and LOEC of nickel were even higher, reaching the magnitude of mg/L [35]. According to the study of the nickel toxicity classification in the tropical marine regions, the 10% nickel hazardous concentration ranged from 7.1 to 41 μg/L, with the average value of 15 μg/L [35,36] was more similar to the range of data in the present study. This concentration is already high compared to the background values of cobalt and nickel in the surface waters of open oceans, and thus, also suggests that there is a greater risk of impacts on pelagic ecosystems as a result of metal releases from polymetallic nodule mining.

Both cobalt and nickel are crucial trace elements in the growth of phytoplankton [37]. The addition of cobalt and nickel in experiments has shown that low levels of cobalt lead to an increase in the cell abundance and chlorophyll *a* content of *S. costatum* and *P. donghaiense*. Conversely, a higher concentration of cobalt resulted in inhibition, which could be attributed to the accumulation rate of heavy metal ions in the cells of *S. costatum* and *P. donghaiense*, as well as the toxicity of heavy metals. In the cobalt addition experiments conducted on *P. donghaiense*, the biomass of the group with a lower cobalt concentration declined due to nutrient deficiencies after an acclimatization period. On the other hand, it appears that the group with a higher concentration of cobalt can sustain the growth of phytoplankton for a longer duration (Figure 1b).

Cobalt and nickel, as heavy metals, can have adverse effects on phytoplankton growth [37,38,39]. This includes the inhibition of the growth process and unfavorable synthesis of cytochromes, as well as reduced photosynthesis [25].

The incorporation of cobalt or nickel enhanced the light absorption of *S. costatum* and *P. donghaiense*, with lower concentrations appearing more favorable than higher concentrations. The pigments diadinoxanthin, alloxanthin, and antheraxanthin act as photoprotective agents, shielding the photosynthetic center from damage via singlet oxygen and harmful radiation [40]. The similar changes observed in diadinoxanthin and alloxanthin suggested that *S. costatum* may need to produce higher amounts of these compounds in the presence of cobalt in order to protect its genetic material from the peroxidation caused by harmful UV radiation. Additionally, elevated levels of cobalt led to a more significant and prolonged threat of peroxidation [34]. The addition of low concentrations of cobalt or nickel accelerated the growth rate of *S. costatum* and *P. donghaiense*, as well as their cellular metabolism, resulting in a higher mortality rate. Hence, in the presence of higher concentrations of cobalt stress or nickel, *S. costatum* cells produced amplified levels of photoprotective pigments, such as diadinoxanthin and alloxanthin, to endure oxidative damage [34]. Cobalt serves as an essential element for phytoplankton growth and can enhance the synthesis rate of photosynthetic pigments such as fucoxanthin, leading to a higher photosynthetic rate efficiency at appropriately low concentrations [39,41].

At appropriate concentrations, cobalt and nickel stimulate intracellular protein and antioxidant enzyme markers [42]. However, high concentrations can lead to inhibited total protein production or cause damage to the protein structure [42]. Lower concentrations of cobalt are more conducive to total protein production compared to high concentrations. Furthermore, lower concentrations of cobalt lead to lower antioxidant enzyme marker activities. Phytoplankton produced supplementary antioxidant enzymes to protect against oxidative stress resulting from environmental changes when exposed to high levels of cobalt stress. By contrast, the levels of total protein and antioxidant enzyme marker activities peaked before decreasing, indicating cell inactivation with stress duration.

Over time, lower concentrations of nickel brought about a boost in protein synthesis in phytoplankton compared to the control group, with a reduction in the activity of each antioxidant enzyme [43]. Meanwhile, the addition of higher nickel concentrations led to a higher intensity of antioxidant enzyme activities, counteracting the effects of peroxidation caused by heavy metals [38]. High concentrations of nickel in the environment resulted in an enhanced resistance to the peroxidative effects of heavy metals by antioxidant enzymes. Conversely, during the process of aging and/or the damage of phytoplankton cells, protein and enzyme activities decreased.

### 4.2. Interaction between Metals

Trace elements are commonly present in minute quantities in the water column, and their impact on phytoplankton primarily revolves around two aspects. Firstly, they act as enzyme cofactors during biochemical reactions, influencing the metabolic processes of phytoplankton [44]. Secondly, they alter the uptake and utilization of other elements, indirectly affecting the growth of phytoplankton. Although a single trace element can have a substantial impact on phytoplankton growth, it is frequently the case that multiple trace elements interact with each other within their effects on phytoplankton growth [45].

Concerning metal absorption, cobalt and nickel exhibited a correlation with iron, manganese, and copper. The coupling of Mn and Co can result from either Co scavenging by Mn oxides or the simultaneous co-oxidation of these elements via a common pathway [46,47,48]. The formation of Mn oxides can also lead to the scavenging of Ni and other bioactive trace metals [49,50]. The differences in cobalt concentrations affected the rate at which phytoplankton took up other metals, with increased cobalt levels aiding iron uptake and heightened nickel concentrations impeding the iron uptake by *S. costatum*. Manganese uptake by *S. costatum* was determined to be higher throughout the entire incubation period, in contrast with the rapid reduction in iron during the initial phase of incubation.

The addition of cobalt resulted in alterations to physiological and ecological indicators, including changes in cell abundance, biomass, pigment composition, and the enzyme activity of phytoplankton. However, it cannot be concluded that these changes were solely due to the individual effect of cobalt, as phytoplankton growth is influenced by the combined impact of various metal elements. An analysis of dissolved metals in the solution indicated that phytoplankton had a notable demand for five trace elements in the following order: Fe > Mn > Cu > Ni > Co. The addition of cobalt at high concentrations resulted in the suppression of algal biomass [51,52], presumably because of the combined effects of cobalt and other metals, such as iron, manganese, and copper. The addition of cobalt at high concentrations resulted in the suppression of the algal biomass. During the initial stages of the experiment, an increased cobalt addition led to an increased iron concentration, which, in turn, resulted in a decreased iron content. During the period when phytoplankton growth was impeded, the concentration of cobalt increased and surpassed that of iron. Varied cobalt concentrations influence the absorption rate of other metals by phytoplankton, whereby increased cobalt concentrations promote the absorption of iron by phytoplankton. Higher cobalt concentrations lead to the increased absorption of iron by phytoplankton, which can explain the low biomass observed in this group with a high cobalt concentration during the initial stage of the study. Furthermore, the reduction process and phytoplankton growth are reliant on iron-containing substances, and the level of iron concentration clearly impacts growth [53]. During the 1–5 day period of the incubation experiment, the presence of metallic iron was observed to have an effect, whilst manganese and copper impacted throughout the long-term experiment, supporting nutrient absorption even when iron uptake was low. Nevertheless, the primary catalyst for the significant phytoplankton growth observed later in the experiment seems to be the result of manganese.

## 5. Conclusions

In this study, *S. costatum* and *P. donghaiense* were used to investigate the effects of cobalt and nickel stress on cell abundance, chlorophyll *a*, pigments, the total protein, and antioxidant enzyme markers, as well as the impact of varying concentrations of cobalt and nickel on the growth and physiological conditions of phytoplankton. The following are the main results:

Firstly, low additions of cobalt or nickel result in phytoplankton exhibiting higher levels of light-absorbing pigments and inactive chlorophyll, as well as lower levels of photoprotective pigments. Additionally, total protein levels increased while antioxidant enzyme activity decreased due to the promotion or inhibition of iron uptake and its interaction with manganese and copper. This ultimately resulted in an increase in cell abundance.

Secondly, high concentrations of cobalt or nickel led to decreased light-absorbing pigments, increased photoprotective pigments, inactive chlorophyll content, lower total protein, and the highest antioxidant enzyme activity in phytoplankton. This is attributed to the efficient uptake/inhibition of iron, which is further accentuated when combined with manganese and copper, ultimately resulting in the inhibition of cell abundance.

Finally, there are similarities in how cobalt and nickel stresses affect phytoplankton. As they experience prolonged stress, both the protein and enzyme activity of the phytoplankton decrease and their cells undergo ageing-induced damage. Additionally, the content of pigments that protect against light and absorb it changes, corresponding to the overall physiological state of the phytoplankton cell.

## Figures and Tables

**Table 1:** The NOEC and LOEC after 120 h for *S. costatum* and *P. donghaiense*. The NOEC and LOEC are in μg/L.

Species	Metals	Parameters	NOEC	LOEC
*S. costatum*	cobalt	cell abundance	14.140	26.077
		chlorophyll *a*	16.251	22.874
		total protein	45.374	66.428
	nickel	cell abundance	3.771	5.505
		chlorophyll *a*	1.720	4.667
		total protein	3.235	5.116
*P. donghaiense*	cobalt	cell abundance	53.271	89.852
		chlorophyll *a*	37.247	66.140
		total protein	42.854	81.237
	nickel	cell abundance	18.428	42.313
		chlorophyll *a*	10.425	17.410
		total protein	22.343	40.174

## Data Availability

Data are available on request to the authors.

## References

[1] Cheng Y., Dai Y., Zhang Y., Yang C., Liu C. (2023). Status and Prospects of the Development of Deep-Sea Polymetallic Nodule-Collecting Technology. Sustainability.

[2] Hund K., La Porta D., Fabregas T.P., Laing T., Drexhage J. (2023). Minerals for Climate Action: The Mineral Intensity of the Clean Energy Transition.

[3] Miller K.A., Thompson K.F., Johnston P., Santillo D. (2018). An Overview of Seabed Mining Including the Current State of Development, Environmental Impacts, and Knowledge Gaps. Front. Mar. Sci..

[4] Christiansen B., Denda A., Christiansen S. (2020). Potential effects of deep seabed mining on pelagic and benthopelagic biota. Mar. Policy.

[5] Cormier R., Londsdale J. (2020). Risk assessment for deep sea mining: An overview of risk. Mar. Policy.

[6] Drazen J.C., Smith C.R., Gjerde K.M., Haddock S.H.D., Carter G.S., Choy C.A., Clark M.R., Dutrieux P., Goetze E., Hauton C. (2020). Midwater ecosystems must be considered when evaluating environmental risks of deep-sea mining. Proc. Natl. Acad. Sci. USA.

[7] Perelman J.N., Firing E., van der Grient J.M.A., Jones B.A., Drazen J.C. (2021). Mesopelagic Scattering Layer Behaviors Across the Clarion-Clipperton Zone: Implications for Deep-Sea Mining. Front. Mar. Sci..

[8] Hauton C., Brown A., Thatje S., Mestre N.C., Bebianno M.J., Martins I., Bettencourt R., Canals M., Sanchez-Vidal A., Shillito B. (2017). Identifying Toxic Impacts of Metals Potentially Released during Deep-Sea Mining—A Synthesis of the Challenges to Quantifying Risk. Front. Mar. Sci..

[9] Ou R., Cai L., Qiu J., Huang H., Ou D., Li W., Lin F., He X., Wang L., Wu R. (2022). Simulation Experiment of Environmental Impact of Deep-Sea Mining: Response of Phytoplankton Community to Polymetallic Nodules and Sediment Enrichment in Surface Water. Toxics.

[10] Zeeshan M., Murugadas A., Ghaskadbi S., Ramaswamy B.R., Akbarsha M.A. (2017). Ecotoxicological assessment of cobalt using Hydra model: ROS, oxidative stress, DNA damage, cell cycle arrest, and apoptosis as mechanisms of toxicity. Environ. Pollut..

[11] Bruland K.W., Donat J.R., Hutchins D.A. (1991). Interactive influences of bioactive trace metals on biological production in oceanic waters. Limnol. Oceanogr..

[12] Ho T.Y., Quigg A., Finkel Z.V., Milligan A.J., Wyman K., Falkowski P.G., Morel F.M. (2003). The elemental composition of some marine phytoplankton. J. Phycol..

[13] Twining B.S., Baines S.B., Bozard J.B., Vogt S., Walker E.A., Nelson D.M. (2011). Metal quotas of plankton in the equatorial Pacific Ocean. Deep. Sea Res. Part II Top. Stud. Oceanogr..

[14] Hong H.-S., Wang M.-H., Huang X.-G., Wang D.-Z. (2009). Effects of macronutrient additions on nickel uptake and distribution in the dinoflagellate Prorocentrum donghaiense Lu. Environ. Pollut..

[15] Niyogi S., Wood C.M. (2004). Biotic Ligand Model, a Flexible Tool for Developing Site-Specific Water Quality Guidelines for Metals. Environ. Sci. Technol..

[16] Twining B.S., Baines S.B. (2013). The trace metal composition of marine phytoplankton. Ann. Rev. Mar. Sci..

[17] Morel F.M.M., Lam P.J., Saito M.A. (2020). Trace Metal Substitution in Marine Phytoplankton. Annu. Rev. Earth Planet. Sci..

[18] Zeng Y., Wang L., Jiang L., Cai X., Li Y. (2015). Joint Toxicity of Lead, Chromium, Cobalt and Nickel to Photobacterium phosphoreum at No Observed Effect Concentration. Bull. Environ. Contam. Toxicol..

[19] Ciğerci İ.H., Ali M.M., Kaygısız Ş.Y., Liman R. (2016). Genotoxicity assessment of cobalt chloride in Eisenia hortensis earthworms coelomocytes by comet assay and micronucleus test. Chemosphere.

[20] Singh N., Bhagat J., Ingole B.S. (2017). Genotoxicity of two heavy metal compounds: Lead nitrate and cobalt chloride in Polychaete Perinereis cultrifera. Environ. Monit. Assess..

[21] Barrio-Parra F., Elío J., De Miguel E., García-González J.E., Izquierdo M., Álvarez R. (2018). Environmental risk assessment of cobalt and manganese from industrial sources in an estuarine system. Environ. Geochem. Health.

[22] Yamatani K., Saito K., Ikezawa Y., Ohnuma H., Sugiyama K., Manaka H., Takahashi K., Sasaki H. (1998). Relative Contribution of Ca^2+^-Dependent Mechanism in Glucagon-Induced Glucose Output from the Liver. Arch. Biochem. Biophys..

[23] Howe P.L., Reichelt-Brushett A.J., Clark M.W. (2014). Investigating lethal and sublethal effects of the trace metals cadmium, cobalt, lead, nickel and zinc on the anemone *Aiptasia pulchella*, a cnidarian representative for ecotoxicology in tropical marine environments. Mar. Freshw. Res..

[24] Sunda W.G. (1989). Trace metal interactions with marine phytoplankton. Biol. Oceanogr..

[25] Sunda W.G. (2012). Feedback interactions between trace metal nutrients and phytoplankton in the ocean. Front. Microbiol..

[26] Guillard R.R.L., Ryther J.H. (1962). Studies of marine planktonic diatoms. I. Cyclotella nana Hustedt and Detonula confervacea Cleve. Can. J. Microbiol..

[27] Parsons T., Maita Y., Lalli C.M., Parsons T. (1984). Amanual of chemical and biological methods for seawater analysis. Biological Oceanographic Processes.

[28] Barlow R., Mantoura R., Gough M., Fileman T. (1993). Pigment signatures of the phytoplankton composition in the northeastern Atlantic during the 1990 spring bloom. Deep Sea Res. Part II Top. Stud. Oceanogr..

[29] Milne A., Landing W., Bizimis M., Morton P. (2010). Determination of Mn, Fe, Co, Ni, Cu, Zn, Cd and Pb in seawater using high resolution magnetic sector inductively coupled mass spectrometry (HR-ICP-MS). Anal. Chim. Acta.

[30] Correggia M., Iorio L.D., Bastianoni A.B., Yücel M., Cordone A., Giovannelli D. (2023). Standard Operating Procedure for the analysis of trace elements in hydrothermal fluids by Inductively Coupled Plasma Mass Spectrometry (ICP-MS). Open Res. Eur..

[31] Organization for Economic Cooperation and Development (OECD) (1984). Guideline for Testing Chemicals. No.201: Alga Growth Inhibition Test.

[32] Dunnett C.W. (1955). A Multiple Comparison Procedure for Comparing Several Treatments with a Control. J. Am. Stat. Assoc..

[33] Manimaran K., Karthikeyan P., Ashokkumar S., Ashok Prabu V., Sampathkumar P. (2012). Effect of copper on growth and enzyme activities of marine diatom, *Odontella mobiliensis*. Bull. Environ. Contam. Toxicol..

[34] Karthikeyan P., Marigoudar S.R., Nagarjuna A., Sharma K.V. (2019). Toxicity assessment of cobalt and selenium on marine diatoms and copepods. Environ. Chem. Ecotoxicol..

[35] Meyer J.S., Lyons-Darden T., Garman E.R., Middleton E.T., Schlekat C.E. (2020). Toxicity of Nanoparticulate Nickel to Aquatic Organisms: Review and Recommendations for Improvement of Toxicity Tests. Environ. Toxicol. Chem..

[36] Gissi F., Stauber J.L., Binet M.T., Golding L.A., Adams M.S., Schlekat C.E., Garman E.R., Jolley D.F. (2016). A review of nickel toxicity to marine and estuarine tropical biota with particular reference to the South East Asian and Melanesian region. Environ. Pollut..

[37] Whitfield M. (2001). Interactions between phytoplankton and trace metals in the ocean. Adv. Mar. Biol..

[38] Guo J.A., Strzepek R., Willis A., Ferderer A., Bach L.T. (2022). Investigating the effect of nickel concentration on phytoplankton growth to assess potential side-effects of ocean alkalinity enhancement. Biogeoscience.

[39] Browning T.J., Rapp I., Schlosser C., Gledhill M., Achterberg E.P., Bracher A., Moigne F.A.C.L. (2018). Influence of Iron, Cobalt, and Vitamin B_12_ Supply on Phytoplankton Growth in the Tropical East Pacific During the 2015 El Niño. Geophys. Res. Lett..

[40] Zigman M., Dubinsky Z., Iluz D. (2012). The Xanthophyll Cycle in Aquatic Phototrophs and Its Role in the Mitigation of Photoinhibition and Photodynamic Damage.

[41] Chmiel R.J., Kell R.M., Rao D., Moran D.M., DiTullio G.R., Saito M.A. (2023). Low cobalt inventories in the Amundsen and Ross seas driven by high demand for labile cobalt uptake among native phytoplankton communities. Biogeoscience.

[42] Osman M.E.H., El-Naggar A.H., El-Sheekh M.M., El-Mazally E.E. (2004). Differential effects of Co^2+^ and Ni^2+^ on protein metabolism in *Scenedesmus obliquus* and *Nitzschia perminuta*. Environ. Toxicol. Pharmacol..

[43] Mccain J.S.P., Bertrand E.M. (2021). Phytoplankton antioxidant systems and their contributions to cellular elemental stoichiometry. Limnol. Oceanogr. Lett..

[44] Rueter J.G., Petersen R.R. (1987). Micronutrient effects on cyanobacterial growth and physiology. N. Z. J. Mar. Freshw. Res..

[45] Zhang Y., Gladyshev V.N. (2010). General trends in trace element utilization revealed by comparative genomic analyses of Co, Cu, Mo, Ni, and Se. J. Biol. Chem..

[46] Tebo B.M., Nealson K.H., Emerson S., Jacobs L. (1984). Microbial mediation of Mn (II) and Co (II) precipitation at the O_2_/H_2_S interfaces in two anoxic fjords. Limnol. Oceanogr..

[47] Lee B.G., Fisher N.S. (1993). Microbially mediated cobalt oxidation in seawater revealed by radiotracer experiments. Limnol. Oceanogr..

[48] Moffett J.W., Ho J. (1996). Oxidation of cobalt and manganese in seawater via a common microbially catalyzed pathway. Geochim. Et Cosmochim. Acta.

[49] Balistrieri L.S., Murray J.W. (1986). The surface chemistry of sediments from the Panama Basin: The influence of Mn oxides on metal adsorption. Geochim. Et Cosmochim. Acta.

[50] Tani Y., Ohashi M., Miyata N., Seyama H., Iwahori K., Soma M. (2004). Sorption of Co (II), Ni (II), and Zn (II) on biogenic manganese oxides produced by a Mn-oxidizing fungus, strain KR21-2. J. Environ. Sci. Health Part A.

[51] Sunda W.G., Huntsman S.A. (1995). Cobalt and zinc interreplacement in marine phytoplankton: Biological and geochemical implications. Limnol. Oceanogr..

[52] Reis L.L.d., Alho L.d.O.G., Abreu C.B.d., Gebara R.C., Mansano A.d.S., Melão M.d.G.G. (2022). Effects of cadmium and cobalt mixtures on growth and photosynthesis of *Raphidocelis subcapitata* (Chlorophyceae). Aquat. Toxicol..

[53] Tada C., Nishimura O., Itayama T., Inamori Y., Matsumura M., Sudo R. (2001). The Influence of Materials Released from Lake Sediment on The Growth of Three Kinds of Algae. Jpn. J. Water Treat. Biol..

